# The accuracy of transcranial Doppler in excluding intracranial hypertension following acute brain injury: a multicenter prospective pilot study

**DOI:** 10.1186/s13054-017-1632-2

**Published:** 2017-02-27

**Authors:** Frank A. Rasulo, Rita Bertuetti, Chiara Robba, Francesco Lusenti, Alfredo Cantoni, Marta Bernini, Alan Girardini, Stefano Calza, Simone Piva, Nazzareno Fagoni, Nicola Latronico

**Affiliations:** 1 Department of Anesthesia, Critical Care and Emergency, Spedali Civili University Hospital, Piazzale Ospedali Civili, 1, 25123 Brescia, Italy; 2 Department of Medical and Surgical Specialties, Radiological Sciences and Public Health, University of Brescia, Brescia, Italy; 3 Department of Neuro Critical Care, Addenbrookes NHS Trust University Hospital, Cambridge, UK; 4 Department of Neuro Critical Care, A. Manzoni Hospital, Lecco, Italy; 5 Department of Anesthesia, Critical Care and Emergency, Circolo Fondazione Macchi Hospital, Varese, Italy; 6 Department of Anesthesia, Critical Care and Emergency, Hospital of Cisanello, Pisa, Italy; 7 Department of Anesthesia, Critical Care and Emergency, Fondazione Poliambulanza Hospital, Brescia, Italy; 8 Unit of Biostatistics, Department of Molecular and Translational Medicine, University of Brescia, Brescia, Italy

**Keywords:** Intracranial pressure, Transcranial Doppler, Intracranial hypertension, Brain injury

## Abstract

**Background:**

Untimely diagnosis of intracranial hypertension may lead to delays in therapy and worsening of outcome. Transcranial Doppler (TCD) detects variations in cerebral blood flow velocity which may correlate with intracranial pressure (ICP). We investigated if intracranial hypertension can be accurately excluded through use of TCD.

**Method:**

This was a multicenter prospective pilot study in patients with acute brain injury requiring invasive ICP (ICPi) monitoring. ICP estimated with TCD (ICPtcd) was compared with ICPi in three separate time frames: immediately before ICPi placement, immediately after ICPi placement, and 3 hours following ICPi positioning. Sensitivity and specificity, and concordance correlation coefficient between ICPi and ICPtcd were calculated. Receiver operating curve (ROC) and the area under the curve (AUC) analyses were estimated after measurement averaging over time.

**Results:**

A total of 38 patients were enrolled, and of these 12 (31.6%) had at least one episode of intracranial hypertension. One hundred fourteen paired measurements of ICPi and ICPtcd were gathered for analysis. With dichotomized ICPi (≤20 mmHg vs >20 mmHg), the sensitivity of ICPtcd was 100%; all measurements with high ICPi (>20 mmHg) also had a high ICPtcd values.

Bland-Altman plot showed an overestimation of 6.2 mmHg (95% CI 5.08–7.30 mmHg) for ICPtcd compared to ICPi. AUC was 96.0% (95% CI 89.8–100%) and the estimated best threshold was at ICPi of 24.8 mmHg corresponding to a sensitivity 100% and a specificity of 91.2%.

**Conclusions:**

This study provides preliminary evidence that ICPtcd may accurately exclude intracranial hypertension in patients with acute brain injury. Future studies with adequate power are needed to confirm this result.

## Background

Brain injury is frequently accompanied by episodes of intracranial hypertension, which is a potentially fatal condition [1–3]. Timely diagnosis through intracranial pressure (ICP) monitoring becomes fundamental in order to guarantee prompt diagnosis and appropriate therapeutic decision-making. Presently, the gold standard for continuous ICP monitoring is invasive measurement through insertion of a catheter within the brain ventricles (EVD) connected to an external pressure transducer [4, 5]. However, this method may be cumbersome, not always available, and accompanied by an elevated complication rate due mostly to infection, hemorrhage, and catheter obstruction [6–9]. Brain intraparenchimal catheters, despite being safer, still require an invasive procedure and cannot be recalibrated once inserted, rendering the measurements prone to imprecision due to zero drift [10–12].

Numerous alternatives to invasive ICP measurement have been proposed in the literature. Although some techniques have potential as screening methods for intracranial hypertension, none have found a valid place within daily clinical practice [13–16]. Among these, methods which use transcranial Doppler (TCD) provide valuable information, as cerebral blood flow velocity has been shown to correlate with ICP [17–22]. In this study, we investigated if ICP estimated by means of TCD (ICPtcd) accurately identifies intracranial hypertension in patients with acute severe brain injury.

## Methods

### Project setting and design

This was a prospective multicenter pilot study and took place between November 2013 and August 2014 in six neurocritical care units (Brescia Spedali Civili University Hospital; Brescia Fondazione Poliambulanza; Pisa Azienda Ospedaliera Cisanello; Lecco Azienda Ospedaliera A. Manzoni; Varese Ospedale di Circolo Fondazione Macchi; Genova Ospedale Galliera). The Brescia University Hospital served as the coordinating center for the study. Ethics approval for all participating sites was obtained from the appropriate regulatory committees. Detailed written information was provided to the family members regarding the study protocol, the scope of research, and the safety of TCD examination. Since all patients had altered consciousness, the ethics committees waived the requirement for consent, as in Italy relatives are not regarded as legal representatives of the patient in the absence of a formal designation [23]. Written informed consent was requested from all surviving patients as soon as they regained their mental competency (NP 1892 – EudraCT: 2014-005482-71).

Patients were included if they were 18 years or older, had sustained acute brain injury and required invasive ICP monitoring within the first 24 hours of ICU admission. They were excluded if they had any one of the following: inaccessible or poor acoustic ultrasound window, a cardiovascular disease causing hemodynamic variations affecting the TCD reading (severe arrhythmia, cardiac valvular stenosis, severe vascular sclerosis), decompressive craniectomy, or any treatment for intracranial hypertension intervening between the invasive ICP (ICPi) and ICPtcd measurements. Patient sedation for ICP bolt placement consisted of bolus followed by continuous infusion of propofol or midazolam, fentanyl, and when necessary, neuromuscular blockade through bolus infusions of atracurium besylate. Mechanical ventilation was targeted to maintain adequate oxygenation (SaO_2_ > 90%) and normocapnia (PaCO2 36–40 mmHg). Intravenous fluids and inotropic support (norepinephrine and/or epinephrine) were provided as appropriate in order to achieve and maintain a sufficient cerebral perfusion pressure (CPP >60 mmHg). General management of the various types of brain injury (traumatic, hemorrhagic, or ischemic), as well as the definition of intracranial hypertension, were in accordance to international guidelines [24–29]. Treatment of intracranial hypertension was based on a protocol-driven strategy which included optimization of arterial blood pressure and volemia, sedation, mild hyperventilation, and infusion of hyperosmolar fluids [30].

### Patient monitoring

Systemic hemodynamic monitoring consisted of invasive arterial blood pressure (ABP) from the radial artery, continuous electrocardiography and pulse oximetry. ICPi was performed either by means of an intraparenchymal fiber-optic transducer (Camino Laboratories, Integra NeuroSciences, San Diego, CA, USA), or a catheter inserted into the brain ventricles and connected to an external pressure transducer and drainage system (Codman, Johnson & Johnson Medical Ltd., Raynham, MA, USA). Cerebral blood flow velocity was assessed using TCD sonography (DWL 2000 Multidop X2, Compumedics DWL, Singen, Germany), and was performed by a selected group of experienced operators in order to reduce inter-operator variability. The insonation technique was standard: a low-frequency pulsed 2 MHz ultrasound probe was placed over the acoustic temporal window for insonation of the M1/M2 section of the middle cerebral artery (MCA) at a depth ranging from 45 to 55 mm [31–33]. The MCAs were insonated bilaterally; however, for ICPtcd measurement the acoustic window ipsilateral to the side of ICP bolt placement was used.

ICPtcd was calculated using the following equations (1 and 2) [21, 22]:

1 $$ ICPtcd = MAP\ \mathit{\hbox{--}}\  CPPe $$

2 $$ CPPe = MAP\ \mathit{\cdotp}\  F V dia\ / F V{m}^{\mathit{\hbox{-}} 1} + 1 4 $$

where MAP represented the mean arterial pressure, CPPe the estimated CPP, FVdia and FVm were, respectively, the diastolic and mean flow velocities, as measured by TCD. The ICPi and MAP readings used for calculations were recorded simultaneously in order to standardize measurements.

### Study design

For each patient enrolled into the study, a total of three ICPtcd measurements were performed, each of which was compared to the corresponding ICPi for concordance. The first ICPtcd measurement (TIME 1) was performed immediately before ICPi placement and was compared with the first ICPi reading once the probe was positioned. The need to reduce the time gap as much as possible between the two readings was motivated by the fact that ICP may be subjected to variations caused by ABP manipulation, cerebrospinal fluid (CSF) leakage during catheter placement and pharmacological treatment or fluctuations due to the evolving underlying brain injury. The second ICPtcd measurement (TIME 2) was performed immediately after insertion of the ICPi probe and compared with the post-insertion ICPi reading. The third ICPtcd measurement (TIME 3) was performed between 2 and 3 hours following the second reading. The reason for this was to avoid any possible variations in systemic and cerebral hemodynamics caused by the ICPi device insertion itself, despite sedation. Therefore, performing the examination more than 2 hours post insertion should reduce the influence of the positioning maneuver on the readings. In accordance with the guidelines present during the study period, intracranial hypertension was defined as an ICP above 20 mmHg, which remained so for at least 10 minutes and was not related to procedural pain [12].

### Statistical analysis

Continuous variables were expressed as means standard deviation (SD) or as medians (interquartile range, IQR) as appropriate, and discrete variables as counts (percentage).

Concordance correlation coefficients were calculated for ICPi and ICPtcd both in patients receiving EVD and intraparenchimal ICP monitoring. These two correlation coefficients were compared by mean of Fisher transformation test, for TIME 1, in order to account for differences due to the possibility of CSF leakage during EVD placement.

Agreement between ICPtcd and ICPi measurements was evaluated both on the continuous raw scale and after categorization based on common usage threshold for ICP (20 mmHg) [34, 35]. Concordance correlation coefficient between ICPi and ICPtcd for repeated measurements was calculated using variance components estimated through linear mixed model, adjusting for ICPi in the three separate time frames described above [36]. A Bland-Altman plot was computed for agreement, assuming constant bias and accounting for linked repeated measures. Linked repeated measures (TIME) were also accounted for variance components and were estimated using Markov chain Monte Carlo [37].

Receiver operating curve (ROC) and the area under the curve (AUC) were estimated after measurement averaging over time. Values of ICPi were dichotomized using a standard reference value of 20 mmHg [34, 35]. Confidence interval for AUC, sensitivity and specificity were computed using bootstrapping (B = 10000) [37]. Youden statistics criterion was used to evaluate the performance of ICPtcd (best combination of sensitivity and specificity) [38]. The sensitivity was expressed as the probability that a patient with high ICPi (>20 mmHg) would also have a high ICPtcd value, and the specificity as the probability that a patient with normal ICPi (≤20 mmHg) would also have a normal ICPtcd value. Best threshold for marker was computed using Youden criterion [38–40].

Sample size for a future study was estimated using the procedure proposed by Flahault et al. and Chu et al., assuming a sensitivity of the test (ICPtcd) of 90%, a prevalence of the disease (intracranial hypertension) equal to 30%, statistical power of 95% and a minimal acceptable lower confidence limit of 10% [41, 42].

R software was used for statistical analysis (version 3.2.5, Free Software Foundation, Inc., Boston, MA, USA).

## Results

From November 2013 to August 2014, a total of 38 patients with acute brain injury were enrolled. Patient demographics, causes of brain injury requiring ICPi monitoring, and types of monitoring techniques are described in Table 1.

**Table 1:** General characteristics and admission diagnoses

Type of brain injury	Number [n]	Age (years) [mean (SD)]	EVD [n]	IP bolt-screw [n]	ICHT [n]
TBI	20	45.1 (12.8)	4/20	16/20	8/20
aSAH	11	61.4 (9)	6/11	5/11	2/11
ICH	7	72.6 (5.7)	0/7	7/7	2/7
TOT cohort	38	57.8 (15.7)	10/38	28/38	12/38

*aSAH* aneurysmal subarachnoid hemorrhage, *EVD* external ventricular drain, *ICH* intracerebral hemorrhage, *ICHT* intracranial hypertension, *TBI* traumatic brain injury

ICP monitoring was initiated in all patients within 24 hours following acute brain injury. EVD was placed in 10 patients, the other 28 received intraparenchimal catheter monitoring. The Fisher transformation analysis of the correlation coefficient for TIME 1 between ICPtcd-IP and ICPtcd-EVD showed no differences (*p* = 0.35), therefore the subsequent analyses were performed without dividing the invasive measurements from IP or EVD.

A total of 114 ICPtcd examinations in three separate time frames were performed in 38 patients, 105 ipsilateral to the ICPi placement and 9 contralateral. The most common reasons for not being able to access the ipsilateral sides were due to an inaccessible acoustic window (60%) and a poor signal (40%). Due to a temporary unavailability of the TCD machine, a transcranial color-coded duplex Doppler was used for ICPtcd measurements in one patient. However, we did not exclude this patient since both ICPi and ICPtcd readings corresponded to values <20 mmHg, and therefore their exclusion would not have modified the results.

As for protocol, during the measurements the PaCO2 in all patients remained within the 36–40 mmHg target range.

**Fig. 1:**
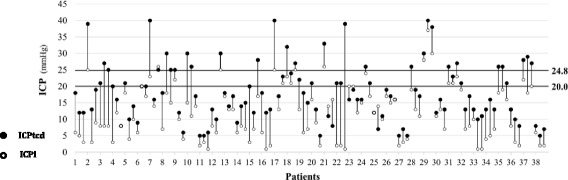
Dichotomized ICP readings, ≤20 mmHg vs >20 mmHg, ≤24.8 mmHg vs >24.8 mmHg. *ICPi* invasive intracranial pressure, *ICPtcd* transcranial Doppler estimation of intracranial pressure

In the 38 patients enrolled, 12 (31.6%) had at least one episode of intracranial hypertension. Of the 114 ICPtcd/ICPi paired readings, elevated ICP was present in 20/114 measurements (17.5%) according to ICPi, and in 41 (35.6%) according to ICPtcd (Table 2). In 6/114 (5%) measurements, the ICPtcd was lower than the corresponding ICPi reading, while in 108/114 (95%) measurements the ICPtcd was higher (Fig. 1). With dichotomized ICPi (≤20 mmHg vs >20 mmHg), the sensitivity of ICPtcd was 100%, all patients with ICPi >20 mmHg also had ICPtcd >20, no false negatives.

**Table 2:** Invasive (ICPi) and transcranial Doppler (ICPtcd) intracranial pressure (ICP) measurements at study times

	ICP values		Number of ICP measurements	
	ICPtcd (mmHg) [mean (SD)]	ICPi (mmHg) [mean (SD)]	ICPtcd >20 mmHg [n (%)]	ICPi >20 mmHg [n (%)]
TIME 1	20.5 (9.1)	13.5 (8.0)	18 (47.3%)	10 (26.3%)
TIME 2	16.7 (7.7)	11.1 (7.8)	10 (26.3%)	4 (10.5%)
TIME 3	17.5 (8.7)	11.5 (8.0)	13 (34.2%)	6 (15.8%)
Overall	18.2 (8.6)	12.0 (7.9)	21 (55.3%)	12 (31.6%)

*TIME1* ICPtcd immediately before ICPi insertion, *TIME 2* ICPtcd immediately after ICPi insertion, *TIME 3* from 2 to 3 h following ICPi insertion

**Fig. 2:**
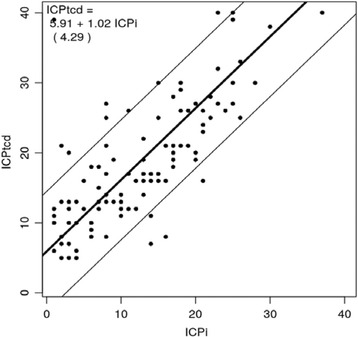
Estimated conversion between ICPtcd and ICPi; slope value = 1.02 (95% CI 0.85–1.36). *ICPi* invasive intracranial pressure, *ICPtcd* transcranial Doppler estimation of intracranial pressure

Figure 2 shows the estimated conversion between ICPtcd and ICPi; the slope value was 1.02 (95% CI 0.85–1.36), which accounts for a 2% increase in bias for unit of ICP (*p* = 0.59), meaning a significant correlation between the measurements provided by the two techniques.

**Fig. 3:**
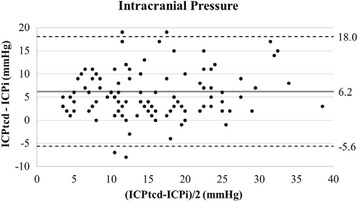
Bland-Altman plot showing mean bias of + 6.2 mmHg for ICPtcd compared to ICPi, with an agreement range from -5.6 to 18 mmHg. *ICPi* invasive intracranial pressure, *ICPtcd* transcranial Doppler estimation of intracranial pressure

The Bland-Altman plot shows an overestimation of 6.2 mmHg (95% CI 5.08–7.30 mmHg) for ICPtcd compared to ICPi, with an agreement range from -5.6 to 18 mmHg (Fig. 3).

**Fig. 4:**
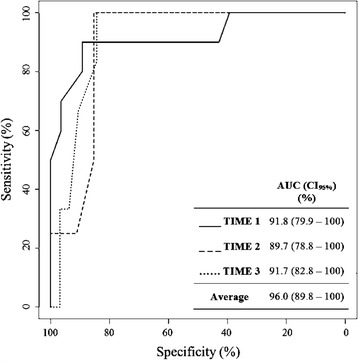
ROC curve analysis for ICPtcd averaged over times (TIME 1, TIME 2, and TIME 3) showing an AUC of 96.0% (95% CI 89.8–100%). The estimated best threshold value was at an ICPi of 24.8 mmHg corresponding to a sensitivity of 100% and a specificity of 91.2%

With a ROC curve analysis for ICPtcd averaged over times (TIME 1, TIME 2, and TIME 3), the AUC was 96.0% (95% CI 89.8–100%) and the estimated best threshold was at ICPi of 24.8 mmHg corresponding to a sensitivity 100% and a specificity of 91.2% (Fig. 4).

The estimated AUC and bootstrapped 95% CI for ROC were estimated at three separate time points (TIME 1, TIME 2, and TIME 3) and the AUC averaged over time. Pairwise comparisons between different AUC did not show any statistically significant difference (1 vs 2, *p* = 0.80; 1 vs 3, *p* = 0.99, 2 vs 3, *p* = 0.78), indicating that ICPtcd estimation of ICPi was time independent.

## Discussion

This is the first prospective multicenter pilot study performed in a cohort of brain-injured patients which showed that ICPtcd had a 100% sensitivity in excluding intracranial hypertension when compared to ICPi. Although the patients were exposed to few episodes of high ICP, this result held true for all values of ICPi above 20 mmHg. The best threshold was at ICPi of 24.8 mmHg corresponding to an ICPtcd sensitivity of 100% and a specificity of 91.2%. ICPtcd was higher than ICPi in the large majority of measurements, which was reflected in the Bland-Altman analysis yielding a mean bias of + 6.2 mmHg. This emphasizes the finding that in patients with acute brain injury recruited in our study, if the ICPtcd was normal, ICPi was certainly normal.

The main goal of our study was to evaluate if ICPtcd could represent a noninvasive screening method to exclude patients without intracranial hypertension and therefore not requiring invasive measurement. There is extensive literature proposing TCD as a tool for noninvasive assessment of ICP [15–22]. In fact, published studies have shown a good concordance between overall ICPi and ICPtcd values. Yet none of these studies have specifically sought to demonstrate that normal ICPtcd can accurately exclude intracranial hypertension. Our results are consistent with a recent multicenter study in 356 traumatic brain injury patients which showed that TCD had a negative predictive value of 98% in excluding neurologic worsening. However, comparison with ICPi was not possible due to the fact that the study enrolled patients with mild to moderate traumatic brain injury. Moreover, the study used the pulsatility index (PI) and diastolic blood flow velocity as TCD parameters to predict neurologic worsening [43]. TCD-derived PI methods are based on observation that ICP and PI are positively correlated during increases of ICP. However, increase in PI is not specific for increase in ICP. In certain situations, such as a drop in CPP, PI presents an increasing trend, which can be related to either increases in ICP or decreases in arterial blood pressure. The same behavior occurs during decrease in PaCO2 or increase in pulsatility of arterial blood pressure waveform. We used the equation for noninvasive measurement of CPP (*CPPe = MAP · FVdia/FVm* ^*-1*^ *+ 14*) proposed by Czosnyka and colleagues based on the fact that specific patterns of TCD waveform, such as a decrease in diastolic flow velocity, reflect impaired cerebral perfusion caused by a decrease in CPP. This formula provides a quantitative assessment of CPP from which ICP can be derived [19–22, 44].

In a recent study, Cardim and colleagues evaluated four methods for noninvasive measurement of ICP; a “black-box” model based on interaction between TCD and arterial blood pressure (nICP_BB); a model based on diastolic flow velocity (nICP_FVd); one based on critical closing pressure (nICP_CrCP); and one on TCD-derived pulsatility index (nICP_PI). The first three methods proved to be the best estimators of measured ICP. We believe these findings strengthen our results, since the method we used was indeed the FVd model. Despite that nICP_FVd had a greater 95% CI for prediction of ICP compared to the other two estimators, it was associated with only a marginally better AUC [45].

Although at present ICPtcd cannot replace ICPi as the gold standard for ICP measurement, this simple and cost-effective method incurs no harm to the patient and provides a method of quickly excluding intracranial hypertension in brain-injured patients in the early phase of hospital admission, when other means are unavailable or contraindicated and when saving time is of paramount importance. In fact, following acute brain injury precious time is frequently lost before adequate cerebral monitoring can be initiated. During triage of polytrauma patients within the emergency department, ICPtcd may be helpful in prioritizing treatment when extracerebral lesions are also involved. Also, on admittance to the emergency department, comatose patients can benefit from early TCD evaluation, which provides valuable information regarding ICP and cerebral perfusion [43]. Admission diastolic flow velocity <25 cm/s and pulsatility index >1.3 in adults and children with head injury have been associated with a poor outcome [43–49]. In a series of 28 severe traumatic brain injury (TBI) patients the authors performed a TCD examination before ICPi monitoring was initiated and identified cerebral hypoperfusion in 46% of patients, which prompted the clinicians to optimize CPP management [49]. Even in the prehospital setting, TCD is feasible and can assist in optimizing early goal-directed therapy [50]. The ICPtcd measurements in our study were performed within 24 hours from brain injury and as soon as possible following ICU admission.

We used a cutoff value of 20 mmHg to define intracranial hypertension, according to the recommendations available at the time the study took place. Current guidelines of the Brain Trauma Foundation indicate that higher ICP values (22 mmHg) should be considered as a threshold [51–54]. We found that ICPtcd sensitivity remained 100% also for all explored values of ICPi above 20 mmHg. Indeed, ROC analyses estimated the best cutoff for sensitivity (100%) and specificity (91.2%) to be at 24.8 mmHg. However, this analysis was based on few measurements of intracranial hypertension episodes, and requires further investigation.

As a pilot study, we also aimed at calculating a sample size for a future larger and more definite trial. In the literature, the prevalence of intracranial hypertension in the categories of acute brain injury taken into consideration in this study [TBI, subarachnoid hemorrhage (SAH), and intracerebral hemorrhage (ICH)], ranged from 36% to 77% [3, 55–57]. Using the calculation method mentioned previously, we estimate a sample size of 490 patients [41, 42].

### Limitations

Some study limitations are worth considering. First, TCD readings were intermittent and not continuous. However, TCD is a noninvasive method which can be rapidly performed and repeated as many times as needed. Second, we enrolled patients with different types of brain injury, including subarachnoid hemorrhage, intracerebral hemorrhage and stroke, for whom ICP thresholds are not well defined. Small sample size precluded us from comparing the diagnostic accuracy of TCD in different diagnostic categories. Third, the study was unblinded; ICPi and MAP were recorded simultaneously in order to reduce the possibility of value selection during the readings. Finally, most of the 114 measurements had ICP <20 mmHg (94/114), therefore the cohort of brain-injured patients was exposed to few episodes of high ICP.

## Conclusions

This prospective multicenter pilot study provides preliminary evidence that ICP estimated with TCD was in line with true ICP in excluding intracranial hypertension. Since the brain-injured patients in our study were exposed to few episodes of high ICP, a study including a greater amount of brain-injured patients with high ICP is warranted.

A large study aiming at enrolling 490 patients is under way (https://www.clinicaltrials.gov - Invasive vs noninvasive Measurement of intracranial PRESSure in brain Injury Trial [IMPRESSIT]).

## Abbreviations

ABP: Arterial blood pressure
AUC: Area under the curve
CPP: Cerebral perfusion pressure
CSF: Cerebrospinal fluid
EVD: External ventricular drain
FV: Flow velocity
ICP: Intracranial pressure
ICPi: Invasive intracranial pressure
ICPtcd: Transcranial Doppler estimation of intracranial pressure
MAP: Mean arterial blood pressure
MCA: Middle cerebral artery
PI: Pulsatility index
ROC: Receiver operating curve
TBI: Traumatic brain injury
TCD: Transcranial Doppler
